# Coverage and determinants of modern contraceptive use in sub-Saharan Africa: further analysis of demographic and health surveys

**DOI:** 10.1186/s12978-022-01332-x

**Published:** 2022-01-21

**Authors:** Isaac Boadu

**Affiliations:** Department of Population, Family and Reproductive Health, University of Ghana, Accra, Ghana

**Keywords:** Contraceptive, Determinants, Coverage, Sub-Saharan Africa

## Abstract

**Background:**

The use of modern contraceptives (MC) in most African countries has been low despite the high fertility rate and unmet need for family planning. This study sought to determine the coverage and determinants of modern contraceptive use among women of reproductive age (15-49 years) in sub-Saharan Africa (SSA).

**Methods:**

Data for the study were obtained from the Demographic and Health Surveys (DHS) conducted between 1995–2020 across 37 SSA countries. Women of reproductive age (15–49 years) was the unit of analysis. Analysis of data was done using STATA version 16 for windows. A bivariate Rao Scott’s Chi-square test of independence was done to determine factors associated with the use of modern contraceptives. Factors that showed significance (p < 0.05) were included in a multilevel logistic regression to determine significant predictors of modern contraceptives. Clustering, stratification and sample weighting were accounted for in the analyses.

**Results:**

The overall prevalence of the use of MC was found to be 22.0%. This ranged from 3.5% in the Central Africa Republic to 49.7% in Namibia. The most common type of MC used were injections (39.4%), condoms (17.5%) and implants (26.5%). Women were less likely to use modern contraceptive if they: had no education (aOR = 0.4, 95% CI 0.38–0.44), had no children (aOR = 0.27–0.42), not told of family planning at a health facility (aOR = 0.69, 95% CI 0.67–0.71), not heard of family planning in the media (aOR = 0.77, 95% CI 0.74–0.79) and being poor (aOR = 0.76, 95% CI 0.73–0.79). On the other hand, women were more likely to use modern contraceptive if they were between the age of 35–39 years (aOR = 1.69, 95% CI 0.73–0.79), married (aOR = 2.66, 95% CI 2.50–2.83), had seven or more children (aOR = 1.27, 95% CI 1.17–0.38), had knowledge of any method of contraceptives (aOR = 303.8, 95% CI 89.9–1027.5) and when field worker visited and talked about family planning (aOR = 1.53, 95% CI 1.39–0.68).

**Conclusion:**

The study showed a low prevalence of modern contraceptive use in sub-Saharan Africa. Findings from the study highlight the need to provide education to women to increase uptake of modern contraceptive and also re-enforce contraceptive interventions to improve women’s health and well-being.

## Plain Language summary

The use of modern contraceptives (MC) to protect against sexually transmitted diseases, unwanted pregnancy and mortality as a result of unsafe abortion is low in many African countries. This study sought to determine the coverage and factors associated with the use of MC among women of child-bearing age in sub-Saharan Africa (SSA). Data for the study were obtained from the Demographic and Health Surveys (DHS) conducted in 37 SSA countries. Interpretation of the data focussed on women of children bearing age (15–49 years). The overall prevalence of the use of MC was found to be 22.0%. This ranged from 3.5% in the Central Africa Republic to 49.7% in Namibia. The most common type of MC used were injections (39.4%), condoms (17.5%) and implants (26.5%). Women were less likely to use MC if they had no education, no children, were not told of family planning at a health facility, had not heard of family planning on the Television, radio, newspaper and were poor. On the other hand, women who were between 35–39 years, were married, had seven or more children, had knowledge of any method of contraceptives and had a field worker visited and talked about family planning were more likely to use modern contraceptives. The study showed a low prevalence of MC use in sub-Saharan Africa. The results from the study is important and emphasize the need to provide education to women of child-bearing age to increase uptake of MC to reduce mortality and improve on women’s health and well-being.

## Background

Among the targets (3.7) in goal 3 of the United Nations sustainable development goals (SDGs) is to ensure universal access to sexual and reproductive healthcare services, including family planning, information and education, and the integration of reproductive health into national strategies and programs [1]. Family planning (FP) has been defined by the World Health Organization (WHO) as a voluntary and informed decision by an individual or couple on the number of children to have and when to have them [2]. It is characterized by the use of contraceptives, either modern or traditional methods. Modern contraceptive methods include male and female sterilization, male and female condoms, depot implants, pills, Lactational Amenorrhea Method (LAM), Intra-Uterine Devices (IUD), and emergency contraception [2]. On the other hand, traditional methods comprise the withdrawal and rhyme method (periodic abstinence) [3]. Of these two methods, modern contraceptive (contraceptive) has been recognized as an effective method for fertility reduction, and are being widely promoted to slow rapid population growth, particularly in developing countries [4, 5].

The use of MC has several benefits. These include birth spacing, reduce unwanted or unintended pregnancies, prevent unsafe abortions, improves maternal health, reduce infant mortality, and prevents sexually transmitted diseases [6]. Identified non-health benefits include expanded education opportunities and empowerment for women, poverty reduction, and ensure sustainable population growth and economic development for countries [7]. It is, therefore, necessary that information on the use of MC is made readily available and accessible to accelerate national efforts of achieving health goals.

Despite these established benefits of family planning, the use of MC is low especially in sub-Saharan Africa countries [3]. Globally, among the 1.9 billion women of reproductive age group (15–49 years) in 2019, 1.1 billion have a need for family planning; of these, 842 million are using contraceptive methods, and 270 million have an unmet need for contraception [2]. Using countries in the family planning 2020 (FP2020) initiative, the average prevalence of modern contraceptive use was estimated to be 23.9% and 28.5% among married women and those engaged in relationships between 2012 and 2017 respectively [3].

In 2012 and 2017 the prevalence of modern contraceptive use among married women or those in relationships in Africa was reported to be low: estimated at 23.9% and 28.5% respectively [3]. In a recent large population-based study to estimate the prevalence and factors associated with modern contraceptive use among women of reproductive age in 20 African countries, Ayampa et al. [8] reported a 26% prevalence of modern contraceptive use with a country-specific variation of 6% in Guinea to 62% in Zimbabwe.

The factors that influence contraceptive practice are multidimensional and have been reported to range from knowledge of contraceptives methods, socio-demographic characteristics (age, education, religion, level of income, marital status, employment), parity, number of living children, source of reproductive information, frequency of antenatal visits, terminated pregnancy, prior HIV testing, residence (rural or urban), literacy, being sexually active, partners communication and approval and fear of side effects of contraceptives [9–16].

Earlier studies on modern contraceptive use in Africa have focused on individual countries [9–11, 13, 17–19]. Very few studies have assessed the prevalence and use of MC across Africa [8, 20–22]. This paucity of information was the drive to this study on the holistic assessment of the prevalence and determinants of modern contraceptive use in the sub-Saharan African region. The study therefore aimed at assessing the coverage and determinants of modern contraceptive use among women of reproductive age (15–49 years) using the available latest demographic and health survey data of sub-Saharan African countries. Findings for this study are important in the global and local context, particularly within the African region to improve maternal and child health outcomes.

## Methods

### Patient and public involvement

Patients were not involved in this study.

### Data source, sampling design and study population

In this study, secondary data of demographic and health survey (DHS) of sub-Saharan Africa countries were used. DHS is a nationally representative household sample survey that evaluates population socio-demographics, maternal and child health, and a variety of health indicators including the use of contraceptives. The DHS is a valuable source of data for studying population health indicators because of its coverage, data quality, and comparability throughout the world. In addition, the sample used is generally representative at the national, regional, and residence (rural and urban) level. DHS sampling is based on a two-stage cluster design approach. In the first stage, there is stratification and proportional allocation of the sample frame. The second stage involves a selection of households per cluster with equal probabilities in a systematic approach. Details of the sampling design and sampling procedure can be found at the DHS program websites (www.dhsprogram.com/methodology/survey-types/DHS).

The available latest demographic and health survey (DHS) conducted in 37 sub-Saharan Africa countries from 1995 to 2020 were included in this study. These countries include Angola, Benin, Burkina Faso, Burundi, Cameroon, Central African Republic, Comoros, Congo, Congo Democratic, Cote d'Ivoire, Eswatini, Ethiopia, Gabon, Gambia, Ghana, Guinea, Kenya, Lesotho, Liberia, Madagascar, Malawi, Mali, Mozambique, Namibia, Niger, Nigeria, Rwanda, Sao Tome and Principle, Senegal, Sierra Leone, South Africa, Sudan, Tanzania, Togo, Uganda, Zambia and, Zimbabwe. Data were downloaded from the DHS programme website (www.dhsprogram.com) after granting permission. The data archive at the DHS website had 38 sub-Saharan Africa countries excluding Ondo State in Nigeria. Chad was not included because of its extremely old data (1990) and was missing most of the independent variables of interest as well as lack of stratification. The unit of analysis in this study was women of reproductive age (15–49 years).

### Definition of variables

#### Outcome variable

The current use of MC by women of reproductive age (15–49 years) was the primary outcome of interest. This was dichotomized as “use of a modern method (coded as “1”) and non-use of modern contraceptive (coded as “0”). Modern contraceptive was described as the use of any of the following contraceptive methods: sterilization (female), intrauterine system (IUD), injectable, implant, tablets, condom (female), standard days method (SDM), emergency contraception, diaphragm, foam/jelly, diaphragm, country-specific modern methods, and other modern contraceptive methods respondent mentioned (including cervical cap, contraceptive sponge, and others) but does not include abortion, menstrual regulation as described in the DHS questionnaire. Traditional methods included periodic abstinence (rhythm, calendar method), withdrawal (coitus interruptus) and country-specific traditional methods of proven effectiveness, and folk methods including locally described methods and/or spiritual methods such as herbs, amulets, gris-gris, etc.

#### Independent variables

The independent variables considered in this study include socio-demographic characteristics such as the age of the respondent (“15–19”, “20–24, 25–29”, “30–34”, “35–39”, “40–44” and “45–49”), age at first birth, recoded (“no birth”, “< 20”, “20–29” and 29+), education (“no education”, “Primary”, “secondary” and “higher”), husband/partners education (“no education”, “Primary”, “secondary” and “higher” “don’t know”) religion, recoded (“Christian”, “Islamic”, “Traditional” and “Other”), marital status, recoded (“Never married” “Married”, “Co-habiting” and “Other”), wealth index, recoded (“poorer/poorest”, “Middle” and “Richer/Richest”), Employment (“working” and “not working”). Others include the number of living children, recoded (“0”, “1–2”, “3–4”, “5–7” and “7+”), source of reproductive information, recoded as media (radio, television, newspaper, text messages, “Yes” and “No”), been told of family planning at a health facility (“Yes” and “No”), place of residence (“rural” or “urban”), the number of sex partners excluding the spouse, recoded (“none”, “1”, “2”, “3+” and “don’t know”) and knowledge of modern contraceptive (“Yes” and “No”), field worker visited and talked about family planning (“Yes” and “No”) and visited health facility in the last 12 months (“Yes” and “No”). These variables were chosen based on previous studies [8, 14, 16, 22].

### Data analyses

Data for this study were analyzed using STATA version 16 for windows. Data were cross-checked for missing data and no response or interviewer error (9 or 99) and were excluded in the analyses. Again, missing data associated with the outcome variable, use of modern contraceptives, were dropped from the analyses. Descriptive statistics were summarised for demographic characteristics and prevalence of the use of MC in each country. A bivariate analysis (Rao Scott’s X^2^) was done to determine the association of socio-demographic characteristics, questions relating to the use of contraceptives, and the outcome variable (use of modern contraceptive). Variables that showed significance in the bivariate analysis were used for the multiple logistic regression analyses. The independent variables were checked for multi-colinearity before the multiple logistic regression. Sample weight was adjusted by dividing the individual women’s sample weight by 1000,000 (v005/10^6^). In all analyses, clustering, stratification, and applied sampling weights were accounted for to reduce bias and to improve on the adjusted estimates and standard errors as recommended in complex survey design analysis. A p < 0.05 was considered statistically significant.

### Ethical approval

This study required no ethical clearance as secondary data were used. However, written permission was sought and was granted from the DHS program before data access.

## Results

### Socio-demographic characteristics of participants

The final sample size of women of reproductive age (15–49 years) for the 37 sub-Saharan Africa countries included in the analyses was 494,285. As of the time of this study, the countries with the latest DHS data were Liberia (2020), Senegal (2019), and Sierra Leone (2019). Central African Republic had the oldest DHS data (see Table 1).

**Table 1:** Country specific sample size and survey year of the DHS

Country	Year	Sample	% Sample
Angola	2015–2016	14,379	2.9
Benin	2017–2018	15,928	3.2
Burkina Faso	2010	17,087	3.5
Burundi	2016–2017	17,269	3.5
Cameroon	2018	13,527	2.7
Central African Republic	1994–1995	5884	1.2
Chad	2014–2015	17,719	3.6
Comoros	2012	5329	1.1
Congo	2011–2012	10,819	2.2
Congo Democratic Republic	2013–2014	18,827	3.8
Cote d'ivoire	2011–2012	10,060	2.0
Eswatini	2006–2007	4987	1.0
Ethiopia	2016	15,683	3.2
Gabon	2012	8422	1.7
Gambia	2013	10,233	2.1
Ghana	2014	9396	1.9
Guinea	2018	10,874	2.2
Kenya	2014	31,079	6.3
Lesotho	2014	6621	1.3
Liberia	2019–2020	8065	1.6
Madagascar	2008–2009	17,375	3.5
Malawi	2015–2016	24,562	5.0
Mali	2018	10,519	2.1
Mozambique	2011	13,745	2.8
Namibia	2013	9176	1.9
Niger	2012	11,160	2.3
Nigeria	2018	41,821	8.5
Rwanda	2014–2015	13,497	2.7
Sao Tome and Principle	2008–2009	2615	0.5
Senegal	2019	8649	1.8
Sierra Leone	2019	15,574	3.2
South Africa	2016	8514	1.7
Tanzania	2015–2016	13,266	2.7
Togo	2013–2014	9480	1.9
Uganda	2016	18,506	3.7
Zambia	2018	13,683	2.8
Zimbabwe	2015	9955	2.0
Total		494,285	100.0

*DHS* Demographic and Health Survey

The mean age of the women was 28.5 ± 0.02 years (95% CI 28.5–28.6%) with 21.2% within the age of 15–19 years. About a third had their highest education in secondary (32.5%) and primary (32.2%). With respect to the educational level of their husbands/partners, 33.3, 27.8, 28.3, and 7.4% had no formal education, primary, secondary, and higher education. Most of the women (60.8%) resided in rural settings and were employed (60.0%). About half (50.9%) were married. With regards to wealth index, 35.9%, were poor/poorest and 44.9% were rich/richest. More than a quarter of the women had 1–2 (28.2%) and 3–4 (28.9%) living children. Women had heard of family planning on the media (42.6%) and had been told of family planning at a health facility (36.1%). Details of socio-demographic and sexual and reproductive characteristics are presented in Table 2.

**Table 2:** Socio-demographic and sexual and reproductive characteristics associated with the use of MC

Variable	Weighted N	Weighted %	%Modern contraceptive use	Rao Scott’s X^2^ (p-value)
Age group [28.5 ± 0.02, (28.5, 28.6)*]				
15–19	104,795	21.2	9.0	1523.6 (< 0.01)
20–24	91,263	18.5	23.9	
25–29	86,634	17.5	27.9	
30–34	71,274	14.4	28.9	
35–39	59,784	12.1	27.6	
40–44	44,301	9.0	23.8	
45–49	36,323	7.4	15.2	
Highest educational level				
No education	147,697	29.9	11.9	1439.4 (< 0.01)
Primary	160,181	32.4	25.6	
Secondary	160,531	32.5	25.9	
Higher	25,925	5.24	32.1	
Highest educational level of husband/partner				
No education	107,539	33.3	12.7	1362.0 (< 0.01)
Primary	89,945	27.8	29.9	
Secondary	91,573	28.3	29.4	
Higher	23,960	7.4	32.1	
Don’t know	10,198	3.2	18.9	
Place of residence				
Urban	193,678	39.2	24.8	245.5 (< 0.01)
Rural	300,696	60.8	20.1	
Employment				
No	190,888	40.0	18.6	510.2 (< 0.01)
Yes	286,403	60.0	23.3	
Marital status				
Never married	137,041	27.7	14.54	674.7 (< 0.01)
Married	251,395	50.9	25.59	
Co-habiting	61,897	12.5	24.09	
Other (widowed/divorced/no longer living with partner)	44,038	8.9	21.32	
Religion				
Christian	289,346	64.6	25.8	1392.7 (< 0.01)
Islamic	133,440	29.8	12.4	
Traditional	6984	1.6	9.4	
Other	18,079	4.0	18.1	
Wealth index				
Poorer/poorest	175,432	35.9	18.0	500.1 (< 0.01)
Middle	93,856	19.2	22.2	
Richer/Richest	219,202	44.9	25.6	
Number of living children				
None	137,905	27.9	9.1	2334.6 (< 0.01)
1–2	149,521	30.2	28.2	
3–4	110,897	22.4	28.9	
5–7	80,183	16.2	23.9	
7+	15,867	3.2	16.9	
Told family planning at a health facility				
No	158,281	63.9	34.0	567.2 (< 0.01)
Yes	89,367	36.1	23.0	
Number of sex partners excluding spouse				
None	391,272	85.5	18.9	1088.8 (< 0.01)
1	60,613	13.3	35.7	
2	5070	1.1	44.0	
3+	575	0.1	48.7	
Don’t know	46	0.01	59.2	
Heard family planning on the media				
No	283,556	57.4	17.4	4321.1 (p < 0.01)
Yes	210,818	42.6	28.0	
Age at first birth (19.3 ± 0.1)*				
No birth	133,134	26.93	9.00	3019 (< 0.01)
< 20	215,240	43.54	26.22	
20–29	138,774	28.07	27.93	
> 29	7225	1.46	19.07	
Knowledge of modern method				
No	30,815	7.2	0.01	9075.9 (< 0.01)
Yes	463,558	92.8	23.42	
Fieldworker visited and talked about family planning				
No	19,861	53.0	11.3	309.8 (< 0.01)
Yes	17,623	47.0	15.6	
Visited health facility last 12 months				
No	229,948	48.1	7.4	4342.4 (< 0.01)
Yes	247,798	51.9	14.0	

*Mean ± Standard error

### Prevalence of modern contraceptive use

**Fig. 1:**
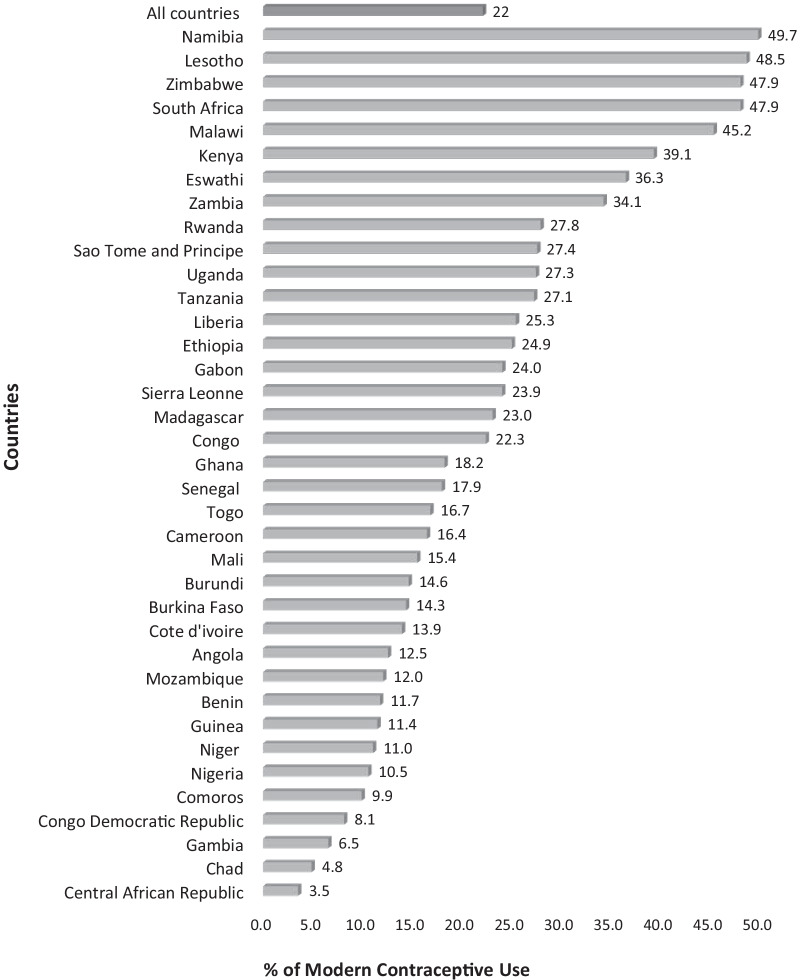
Prevalence of MC use among women (15–49) years in sub-Saharan Africa. The pooled prevalence of modern contraceptive use was 22.0% (95% CI 21.8–22.2%). Coverage varied considerably across countries, ranging from the highest, 49.7% (95% CI 48.4–51.1%) in Namibia to lowest, 3.5% (95% CI 3.0–4.1%) in Central Africa Republic

**Fig. 2:**
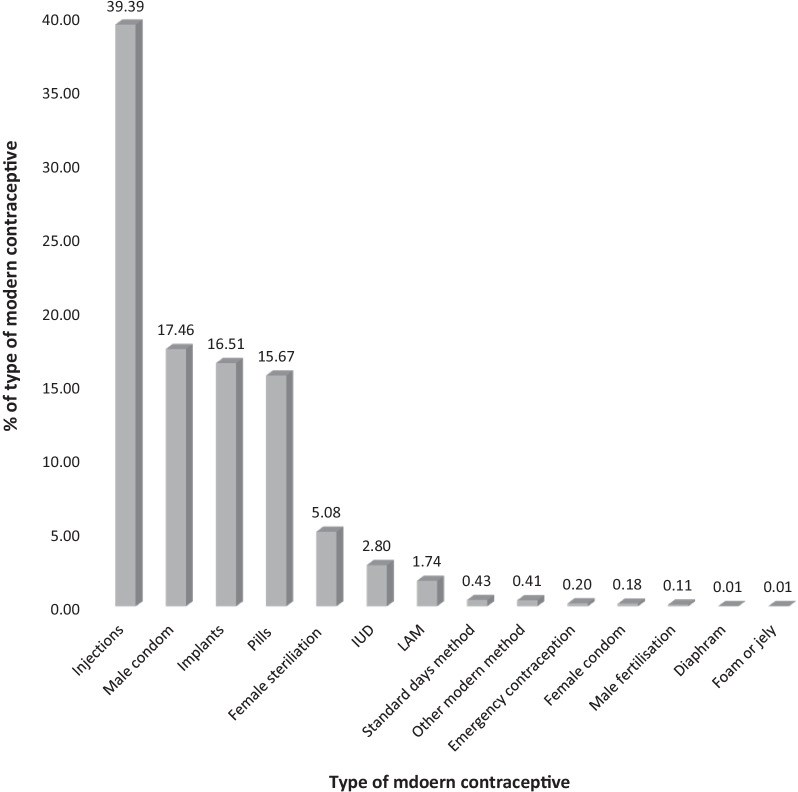
Type of modern contraceptive used. The most commonly used family planning method (modern contraceptives) were injections (39.4%), male condoms (17.5%), implants (16.5%), and pills (15.7%)

The pooled prevalence of modern contraceptive use was 22.0% (95% CI 21.8–22.2%). Coverage varied considerably across countries, ranging from the highest, 49.7% (95% CI 48.4–51.1%) in Namibia to lowest, 3.5% (95% CI 3.0–4.1%) in Central Africa Republic. Other countries that had high prevalence of MC use were Lesotho, 48.5% (95% CI 21.8–22.2%), Zimbabwe, 47.9% (95% CI 46.5–49.2%), South Africa, 47.9% (95% CI 46.2–49.5%), Malawi, 45.2% (95% CI 44.2–46.1%) and Kenya, 39.1% (95% CI 38.2–40.0%) (Fig. 1). The most commonly used family planning method (modern contraceptives) were injections (39.4%), male condoms (17.5%), implants (16.5%), and pills (15.7%) (Fig. 2).

### Factors associated with modern contraceptive use

In a bivariate analysis (Rao Scott’s X^2^)**,** all the socio-demographic characteristics used in the study (educational level, place of residence, religion, employment status, marital status, and wealth index), as well as the reproductive and sexual characteristics, were significantly associated with the use of modern contraceptive (p < 0.05).

In a multilevel logistic regression model (Table 3), women aged 35–39 years had higher odds [aOR = 1.69, 95% CI (1.58–1.80)] of using MC compared with women aged 15–19 years. Women with no formal education were less likely to use MC [aOR = 0.4, 95% CI (0.38–0.44)] compared with women with higher education. Living in rural areas was less associated [aOR = 0.76; (0.72–0.89)] with the use of MC compared with living in urban areas. Women who were Christians were more likely to use MC than other types of religion (aOR = 1.3, 95% CI 1.17–1.38). Being poor/poorest [aOR = 0.76, 95% CI (0.73–0.79)] and belonging to the middle-class wealth index [aOR = 0.98, 95% CI (0.80–1.20)] were less associated with the use of modern contraceptive use compared with rich or richest women. Women with 5–7 children were more likely to use MC [aOR = 1.27, 95% CI (1.17–1.38)].

**Table 3:** Factors associated with the use of modern contraceptive use among women (15–49 years)

	aOR (95% CI)
Age group (years)	
15–19	1.24 (1.13–1.35)**
20–24	1.66 (1.55–1.79)**
25–29	1.68 (1.56–1.79)**
30–34	1.67 (1.56–1.78)**
35–39	1.69 (1.58–1.80)**
40–44	1.58 (1.48–1.70)**
45–49	1
Education	
No education	0.40 (0.38–0.44)**
Primary	0.82 (0.77–0.88)**
Secondary	0.89 (0.84–0.95)*
Higher	1
Highest educational level of husband/partner	
No education	1
Primary	1.33 (1.17–1.50)**
Secondary	1.26 (1.10–1.43)*
Higher	1.30 (1.10–1.54)*
Don’t know	1.24 (0.93–1.68)
Marital status	
Never in union	1
Married	2.66 (2.50–2.83)**
Living with partner	1.68 (1.57–1.80)**
Other (widowed/divorced)	0.93 (0.87–0.99)*
Place of residence	
Urban	1
Rural	0.76 (0.72–0.89)**
Employment	
No	1
Yes	1.03 (0.99–1.06)
Religion	
Christian	1.3 (1.17–1.38)**
Islamic	0.66 (0.60–0.72)**
Traditional	0.46 (0.39–0.55)*
Other	1
Wealth Index	
Poorer/poorest	0.76 (0.73–0.79)**
Middle	0.89 (0.85–0.93)**
Richer/Richest	1
Age at first birth	
No birth	1
< 20	0.98 (0.80–1.20)
20–29	0.88 (0.72–1.08)
29+	0.56 (0.45–0.71)**
Number of living children	
None	0.34 (0.27–0.42)**
1–2	1.03 (0.94–1.13)
3–4	1.27 (1.17–1.39)**
5–7	1.27 (1.17–1.38)**
7+	1
Number of sex partner (s) excluding spouse	
None	1
1	4.22 (4.00–4.45)**
2	7.31 (6.34–8.43)**
3+	9.57 (6.62–13.84)**
Told of family planning at a health facility	
No	0.69 (0.67–0.71)**
Yes	1.00
Heard of family planning on Media	
No	0.77 (0.74–0.79)**
Yes	1.00
Knowledge of modern method	
No	1
Yes	303.8 (89.9–1027.5)**
Fieldworker visited and talked about family planning	
No	1
Yes	1.53 (1.39–1.68)**
Visited health facility last 12 months	
No	1
Yes	1.26 (1.16–1.36)**

Multiple logistic regression: Dependent variable—use of modern contraceptive (use/non-use)

aOR: Adjusted odds ratio; 95% CI: 95% confidence interval. 1-Reference category

**p < 0.001; *p < 0.01

Having more (3+) multiple sexual partners excluding spouse was associated with higher odds [aOR = 9.57, 95% CI (6.62–13.84)] of the use of MC compared with having no multiple sexual partners. Women who were not told of family planning at a health facility were less likely [aOR = 0.69, 95% CI (0.67–0.71)] to use MC compared with women who had heard family planning from a health facility. Knowing any modern method of contraceptive was 303.8 times associated with the use of MC [aOR = 303.8, 95% CI (89.9–1027.5)] than those who did not know any method. Women who had not heard of family planning from television, radio, and text messages (media as a source of information) were less likely to use MC [aOR = 0.77, 95% CI (0.74–0.79)] compared with those who have heard of family planning from the media.

## Discussion

Family planning is one of the investments that can be made to help achieve most of the United Nation’s sustainable development goals especially poverty reduction, quality education, decent work and economic growth, and good health and well-being. The use of MC is a safe and effective method to regulate fertility and ensure the well-being of women of reproductive age. Coverage and factors associated with the use of MC vary across the globe including sub-Saharan Africa countries.

In this study, the pooled prevalence of MC use was low, 22.0%; with country-specific variations. Namibia recorded the highest reproductive women using MC (49.7%) with the least in Central Africa Republic (3.5%). This finding is similar to a recent study using secondary data of DHS in 29 sub-Saharan Africa countries on predictors among adolescent and young women, where Ahenkorah [22] reported a prevalence of 24.7%. Again, the finding is in agreement with a study by Apanga et al. [8] who used data from multiple indicator cluster surveys across 20 Africa countries. The authors reported an overall prevalence of 26% ranging from 6% in Guinea and 62% in Zimbabwe. In addition, this finding is also consistent with country-specific DHS studies [9, 16, 23–27].

The most common type of contraceptives used were injections (39.4%), condoms (17.5%), and implants (26.5%). Earlier studies have reported similar findings [9, 12]. Factors associated with modern contraceptive use include age, women’s educational level, educational level of husband/partner, place of residence, employment, marital status, wealth index, number of living children, been told of family planning at a health facility, number of sex partners excluding the spouse, heard family planning on the media (television, radio, newspaper, text messages), knowledge of modern method and a visit by a health worker to discuss family planning.

In this present study, younger aged women (15–19 years) were less likely to use MC compared to older old women (35–39 years). This could be due to the fact that, these old women were having more children and would want to limit or space the number of pregnancies than the younger women who may have few or no children. This was confirmed in the findings where women with more children were less likely to use modern contraceptives. This finding is in line with previous studies [12, 28] but contradicts the findings of these studies [22, 29] where younger women were more likely to use modern contraceptives.

In most African countries family planning is part of integrated health care. Health workers continue to play a major role in encouraging the use of MC by providing couples with the knowledge that enables them to make informed reproductive decisions including the use of MC [30]. Findings from this study indicate that women who had received family planning information from health workers were more likely to use MC than those who did not. This is in agreement with the study by Kebede and colleagues [12].

Women residing in rural areas were less likely to use MC than those in urban settings. This could be due to accessibility, availability, and lack of information on the use of MC in these settings. Consistent with this finding is the study by Kebede et al., Ontiri et al. and Mahmood and Ringheim [12, 19, 31].

The media serves as a source of information and is mostly used to provide vital information to the public. In this study, women who reported receiving information from the media (television, radio, newspaper, text messages) or exposed to the media were more likely to use modern contraceptives. This is similar to earlier studies that reported on the strong influence and association of the media and uptake of MC [32, 33].

Having adequate reproductive knowledge helps with better decisions. A finding from this study is that knowledge of any method of contraceptives was significantly associated with the use of modern contraceptives. This is in agreement with previous studies [12, 34]. It is therefore imperative that much reproductive information is provided to women to increase their knowledge to guide good reproductive decisions to improve their health and well-being.

## Strength and limitation of the study

The strength of this study is the large population-based sample size used in the study which increased the power of the study. This enables the generalizability of the findings of modern contraceptive use among women of reproductive age. However, the study was limited by the use of secondary data restricting study variables.

## Conclusion

There was a low pooled prevalence (22.0%) of the use of MC across the 37 countries used in sub-Saharan Africa. This showed a considerable variation from as low as 3.5% in the Central Africa Republic to 49.7% in Namibia. The most common type of contraceptives used were injections (39.4%), condoms (17.5%), and implants (26.5%). Socio-demographic, sexual characteristics factors were found to be associated with the use of modern contraceptives. The low prevalence of modern contraceptive use recorded in this study infers that there should be more education particularly in the health facility and the media to increase knowledge and uptake of MC use among women of reproductive age.

## Data Availability

All datasets and materials supporting our findings are available from the DHS program website. I acknowledge all reviewers for their useful comments.

## Abbreviations

DHS: Demographic and Health Survey
SSA: Sub-Saharan Africa
MC: Modern contraceptive
aOR: Adjusted odds ratio
LAM: Lactational Amenorrhea Method
IUD: Intra-Uterine Devices
FP: Family planning
